# Supplementary material for: The Systems Biology Research Tool: evolvable open-source software
Source: BMC Syst Biol. 2008 Jun 29;2:55. doi: 10.1186/1752-0509-2-55 (PMC2446383; doi:10.1186/1752-0509-2-55)
Supplement: Additional file 1 — SBRT Archive. An archive of the current version of the Systems Biology Research Tool. [file 1752-0509-2-55-S1.zip › sbrt-1.4.0/doc/developers_guide/api/sbrt/kernel/math/algebra/package-frame.html]

sbrt.kernel.math.algebra


sbrt.kernel.math.algebra

Interfaces 
  
*DoubleMatrix*
  
*DoubleMatrixBuilder*
  
*LinearSystemSolution*
  
*LinearSystemSolver*
  
*Matrix*
  
*MatrixBuilder*
  
*SmdMatrix*
  
*SparseDoubleMatrix*

Classes 
  
AbstractMdMatrix
  
AbstractSmdMatrix
  
DoubleMatrixWrap
  
HashLinearSystemSolution
  
HashMatrix
  
IndexPair
  
MatrixElement
  
MatrixMultiplier
  
MatrixTransposer
  
MatrixWrap
  
RowOrTreeMatrix
  
SparseDoubleMatrixWrap
  
TreeMatrix

Exceptions 
  
IllegalFreeVariableException
